# Crystal-Orientation-Dependent Oxygen Exchange Kinetics on Mixed Conducting Thin-Film Surfaces Investigated by *In Situ* Studies

**DOI:** 10.1021/acsaem.3c00870

**Published:** 2023-06-13

**Authors:** Matthäus Siebenhofer, Christoph Riedl, Andreas Nenning, Sergej Raznjevic, Felix Fellner, Werner Artner, Zaoli Zhang, Christoph Rameshan, Jürgen Fleig, Markus Kubicek

**Affiliations:** † Institute of Chemical Technologies and Analytics, Vienna University of Technology, Vienna 1060, Austria; ‡ Centre for Electrochemistry and Surface Technology (CEST), Wiener Neustadt 2700, Austria; § Erich Schmid Institute of Materials Science, Austrian Academy of Sciences, Leoben 8700, Austria; ∥ X-Ray Center, Vienna University of Technology, Vienna 1060, Austria; ⊥ Chair of Physical Chemistry, Montanuniversität Leoben, Leoben 8700, Austria

**Keywords:** oxygen exchange, mixed conducting oxides, work function, pulsed laser deposition, sulfate adsorbates

## Abstract

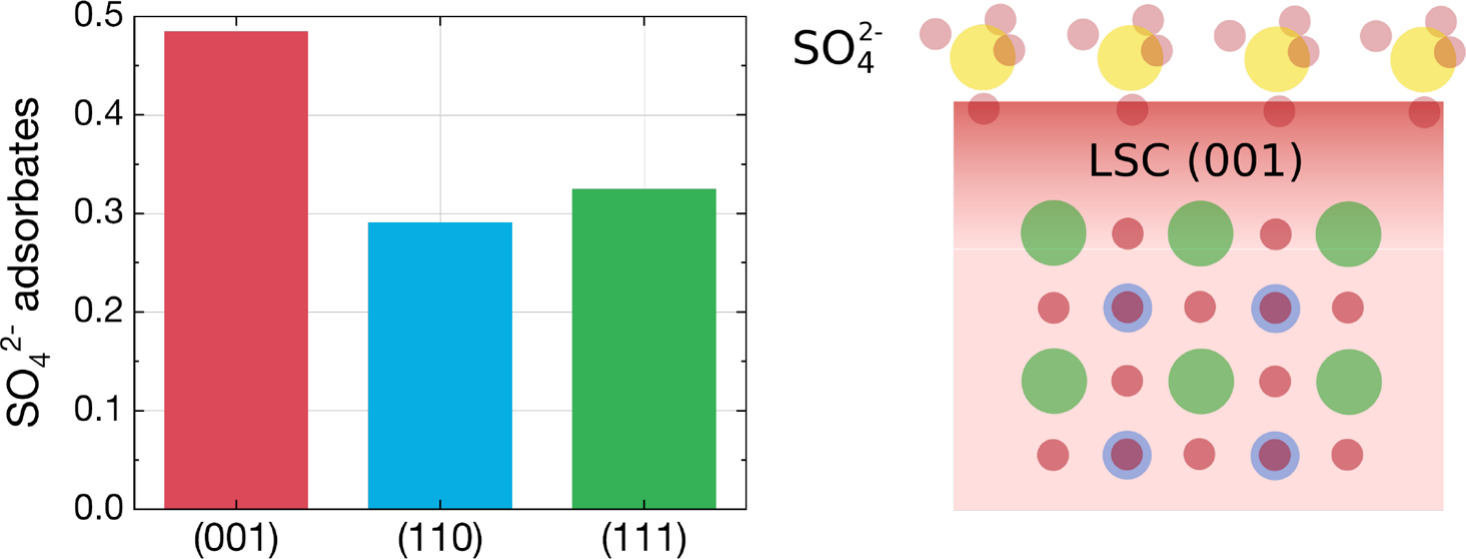

The oxygen exchange kinetics and the surface chemistry of epitaxially grown, dense La_0.6_Sr_0.4_CoO_3−δ_ (LSC) thin films in three different orientations, (001), (110), and (111), were investigated by means of *in situ* impedance spectroscopy during pulsed laser deposition (i-PLD) and near-ambient-pressure X-ray photoelectron spectroscopy (NAP-XPS). i-PLD measurements showed that pristine LSC surfaces exhibit very fast surface exchange kinetics but revealed no significant differences between the specific orientations. However, as soon as the surfaces were in contact with acidic, gaseous impurities, such as S-containing compounds in nominally pure measurement atmospheres, NAP-XPS measurements revealed that the (001) orientation is substantially more susceptible to the formation of sulfate adsorbates and a concomitant performance decrease. This result is further substantiated by a stronger increase of the work function on (001)-oriented LSC surfaces upon sulfate adsorbate formation and by a faster performance degradation of these surfaces in *ex situ* measurement setups. This phenomenon has potentially gone unnoticed in the discussion of the interplay between the crystal orientation and the oxygen exchange kinetics and might have far-reaching implications for real solid oxide cell electrodes, where porous materials exhibit a wide variety of differently oriented and reconstructed surfaces.

## Introduction

The design of catalytically active and degradation-resilient air electrode materials for the oxygen exchange reaction (OER) in solid oxide fuel and electrolysis cells (SOFCs and SOECs) is a key challenge on the way toward a broad application of this promising energy conversion technology.^1,2^ Under operating conditions, two main degradation drivers are observed for such materials: (i) chemical and morphological changes of the materials, for example, by cation segregation (e.g., for Sr-containing perovskites),^3−6^ and (ii) performance degradation due to detrimental impurities from the surroundings (such as S and CO_2_ content in the gas phase, as well as Cr from interconnects or Si and B from sealing materials).^7−13^ Several attempts have been made to mitigate the effects of both degradation drivers first and foremost by modifications of the cathode surface with other materials.^14−16^ In addition, the effects of surface morphology and strain on the kinetics and the degradation of air electrode materials have been investigated in great detail.^17−19^

Commercial solid oxide cells often rely on porous, polycrystalline cathode materials, applied by screen printing or spraying methods.^20,21^ Scientific studies, on the other hand, are often performed on model systems, such as thin-film electrodes.^22−25^ A central difference between the two, apart from their morphological properties, is the different distribution of crystal orientations. While polycrystalline bulk samples usually exhibit a variety of different orientations,^26^ the choice of the substrates largely controls the preferred orientation or even the epitaxial growth of a thin-film electrode.^27,28^ In the case of solid oxide cells, the choice of single-crystalline electrolyte materials as substrates for thin-film deposition is limited. Different crystal orientations are easily available for YSZ, while LSGM or GDC substrates in well-defined orientations require special preparation and are very expensive. Thus, systematic studies of the effects of crystal orientations on the oxygen exchange kinetics (especially for perovskites) are difficult, at least by electrical measurements, and the understanding of the underlying mechanics is limited.

The effect of crystal orientation on the OER rates has been investigated for various materials, such as SrTiO_3_,^29,30^ Ruddlesden–Popper class materials (La_1.85_Sr_0.15_CuO_4−δ_ and Nd_2_NiO_4+δ_)^31,32^ or La,Sr-based perovskites (La_1–*x*_Sr_*x*_Co_1–*y*_Fe_*y*_O_3−δ_ and La_1–*x*_Sr_*x*_MnO_3−δ_).^33−37^ In general, all studies agree that (001) surfaces exhibit the lowest catalytic activity. For other orientations (usually (110)- and (111)-oriented surfaces were investigated), the different studies yielded ambivalent results and various underlying reasons for different kinetics on specific crystal orientations, in particular the importance of the surface chemistry (reconstructions, vacancy concentrations, O_2_ adsorption energies, and polarity), as well as the impact of strain and morphology have been emphasized.

In this work, La_0.6_Sr_0.4_CoO_3−δ_ (LSC) thin films were grown by pulsed laser deposition (PLD) in three different orientations, (001), (110) and (111), and their electrochemical properties as well as their surface chemistry were investigated and compared. *In situ* impedance spectroscopy during pulsed laser deposition (i-PLD), a technique that enables investigations in very clean environments, was employed to assess the catalytic activity of the three orientations in their pristine state. Near-ambient-pressure X-ray photoelectron spectroscopy (NAP-XPS) measurements of LSC surfaces and *ex situ* impedance spectroscopy were performed to obtain insight into the interplay between degradation of the OER kinetics and different crystal orientations. The results confirm that acidic adsorbates, in particular, sulfate adsorbates, originating from omnipresent trace impurities (e.g., ≈0.5 ppmv sulfur compounds in measurement atmospheres fed from 5.0 purity (99.999%) synthetic air),^11^ strongly inhibit the oxygen exchange kinetics of pristine LSC and play a decisive role in the observable catalytic activity of differently oriented LSC surfaces.

## Experimental Section

### Sample Preparation

LSC thin films were deposited by PLD on La_0.95_Sr_0.05_Ga_0.95_Mg_0.05_O_3−δ_ (LSGM) single crystals in (001), (110), and (111) orientations, as well as on yttria-stabilized zirconia (YSZ) single crystals with a Gd_0.2_Ce_0.8_O_2−δ_ (GDC) buffer layer in (001) and (111) orientations. YSZ single crystals (9.5 mol% Y_2_O_3_,) were purchased from Crystec GmbH, Germany, and LSGM single crystals were grown by the Czochralski technique (details in ref (38)) and cut into smaller specimens by Surfacenet GmbH, Germany. Before electrode deposition, the current collecting Ti/Pt grids (5/100 nm, 25 μm holes, and 5 μm grid) were prepared by lift-off photolithography and metal sputtering (BalTec MED 020, Leica Microsystems GmbH, Germany) on both sides of the single crystals. PLD was done with a KrF (λ = 248 nm) excimer laser (Lambda physics, COMPex Pro 201) and a laser fluence of 1.1 J/cm^2^. As counter electrodes, 300 nm nanoporous LSC thin films were deposited on one side of the single crystals at 450 °C substrate temperature, 0.4 mbar O_2_, a substrate–target distance of 5.0 cm, and a laser frequency of 5 Hz. This parameter set facilitates the deposition of nanoporous columnar thin films with large inner surface area and very fast oxygen exchange kinetics.^5^ Working electrode depositions were performed at different deposition parameters: the substrate temperature amounted to 500 °C for LSGM or 600 °C for YSZ|GDC substrates, the background pressure was set to 0.04 mbar O_2_, and a substrate–target distance of 6.0 cm and a frequency of 2 Hz were used. Growth rates were determined by film thickness measurements with a profilometer.

### Electrochemical Characterization

i-PLD was performed with a custom-made setup inside the PLD chamber.^40^ The bottom Ti/Pt grid was contacted with a brushed Pt electrode on the heating stage, and the top grid was contacted with a Pt/Ir needle. The temperature during i-PLD was controlled by evaluating the ohmic offset in an across-plane impedance measurement. This offset resistance includes contributions from wiring and grid resistances, which were determined beforehand, as well as from thermally activated ionic conduction through the electrolyte substrates. With the known conductivity–temperature relationships for YSZ and LSGM,^38,41^ this technique allows for a very exact temperature measurement during the PLD process. Impedance measurements were conducted with an Alpha-A High Performance Frequency Analyzer and Electrochemical Test Station POT/GAL 30 V/2A setup by Novocontrol Technologies in the frequency regime from 10^6^ to 10^–1^ Hz with an alternating-current (ac) voltage of 20 mV root mean square (RMS). *Ex situ* measurements in synthetic air (5.0 purity, Messer, Austria) were performed with the same setup by Novocontrol Technologies and a measurement setup in a tube furnace. The temperature was measured with a type S thermocouple, positioned 1 cm next to the sample.

### Structural Characterization

#### X-ray Diffraction (XRD)

Samples were investigated by XRD as well as by atomic force microscopy (AFM). θ–2θ scans were performed in an Empyrean X-ray diffractometer (Malvern Panalytical) equipped with a hybrid monochromator.

#### High-Resolution Transmission Electron Microscopy (HRTEM)

HRTEM analysis was performed using a JEOL 2100F microscope operated at 200 kV and equipped with an image-side corrector. Samples were prepared by two techniques. The (001)-oriented sample was prepared by the conventional method, which includes grinding, dimpling, and finally Ar-ion milling. The (110)- and (111)-oriented samples were prepared by the lift-out technique on a Thermo Fisher Scios 2 DualBeam focused ion beam/scanning electron microscope with a Ga-ion beam operating at 30 kV accelerating voltage. For the final thinning and polishing, the voltage was successively reduced to 5 and 2 kV. Geometrical phase analysis (GPA) was used to determine the relative difference of the out-of-plane lattice parameter between the substrate and thin film.

### Surface Characterization

AFM measurements were performed in tapping mode with a Nanoscope V multimode setup (Bruker) over a scan area of 1 × 1 μm^2^. Images were processed with *Gwyddion*.^42^ X-ray photoelectron spectroscopy was performed to investigate the surface chemistry of differently oriented LSC thin films with a laboratory-based setup using a PHOIBOS NAP photoelectron analyzer (SPECS, Germany) and a monochromated Al Kα XR 50 MF (microfocus) X-ray source. The samples were mounted on a custom sample holder with a 4.5 × 4.5 mm^2^ hole for laser heating with a near-infrared diode laser.^43^ Samples were fixed with Pt–Ir needles, which were also used as electrical contacts. The sample temperature was checked with a pyrometer. At lower temperatures (400 °C), precise temperature control is possible by evaluating the high-frequency ohmic offset of an impedance curve. This was used to calibrate the sample emissivity. XPS spectra were recorded in 8 × 10^–6^ mbar O_2_ (5.0 purity; Messer GmbH, Austria) at 600 °C. Impedance spectra were again measured with an Alpha-A High Performance Frequency Analyzer and Electrochemical Test Station POT/GAL 30 V/2A setup (Novocontrol Technologies, Germany). To cover the surface with sulfate adsorbates, the pressure in the XPS chamber was increased to 1 mbar O_2_. XPS spectra were recorded at an analyzer pass energy of 30 eV, and for work function investigations, an analyzer pass energy of 5 eV and an energy step width of 0.02 eV were used. A sample bias of −20 V was applied to accelerate very low energy electrons toward the analyzer. Because of the low electron energy and the long path in the NAP analyzer, a precisely tuned active magnetic shielding was mandatory.

## Results

### Sample Characterization

#### XRD

**Figure 1:**
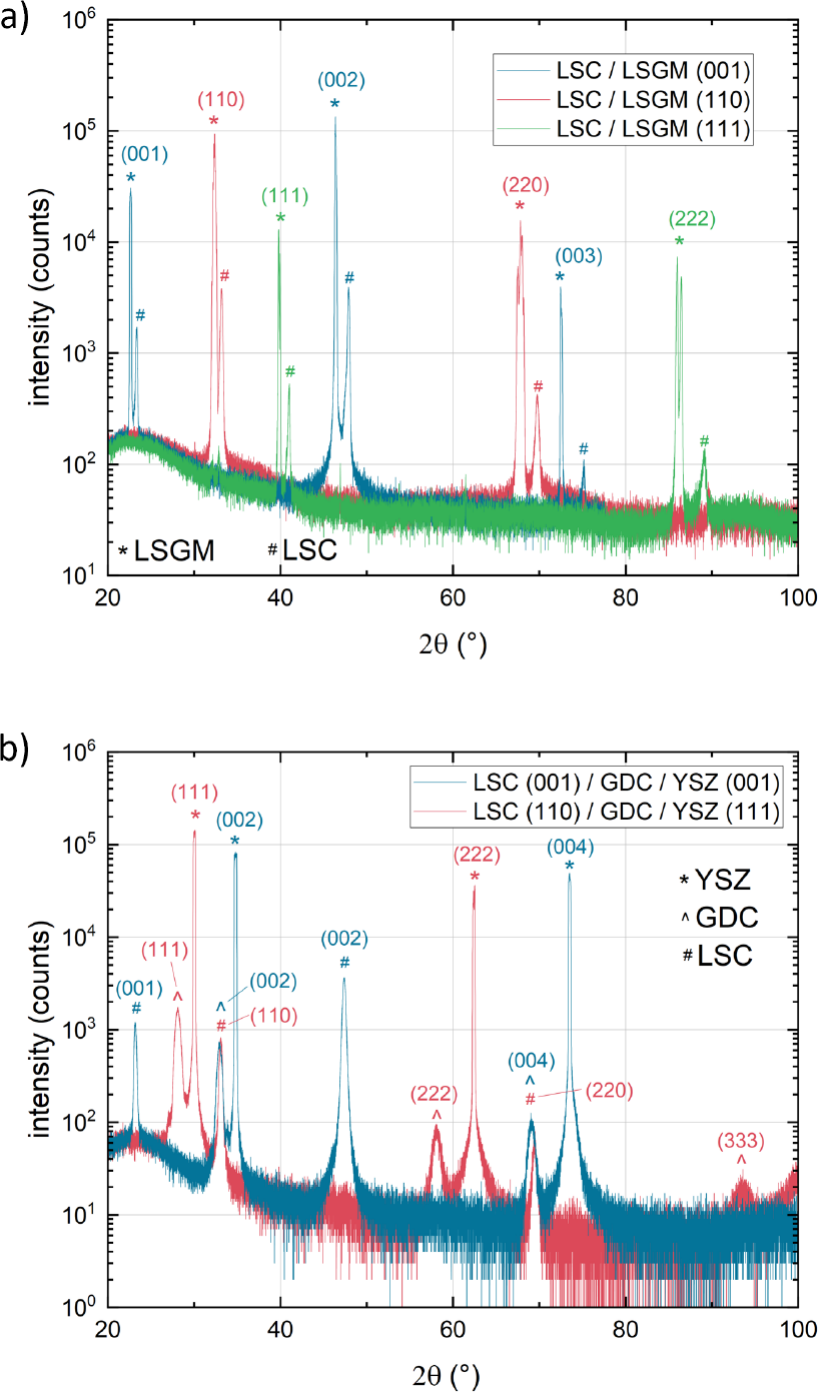
(a) θ–2θ scans of three samples of LSC grown on LSGM single crystals in the orientations (001), (110), and (111). (b) θ–2θ scans of two samples of LSC grown on YSZ single crystals in the orientations (001) and (111) with a GDC buffer layer.

The XRD measurements of 120 nm LSC thin films on LSGM (001), (110), and (111) substrates are shown in Figure 1a. The diffractograms show a clear relationship between the substrate and film orientation. The same holds for the diffractograms of LSC thin films grown on YSZ (001) and (111) with a GDC buffer layer (Figure 1b). While the oriented growth of LSC on LSGM is not surprising due to the similar crystal structure, oriented growth of LSC on YSZ is only facilitated by the GDC buffer layer.^37,44^ The resulting out-of-plane lattice parameter of LSC, *a*_⊥_, and the out-of-plane strain (compared to 3.84 Å for polycrystalline LSC with mixed orientations grown directly on YSZ (001)^45^) are shown in Table 1.

**Table 1:** Out-of-Plane Lattice Parameters of Different LSC Thin Films and the Corresponding Out-of-Plane Strain^a^

	*a*_⊥_(XRD) (Å)	strain_⊥_ (%)
LSC(001)/LSGM	3.81	0.8
LSC(110)/LSGM	2.70	0.7
LSC(111)/LSGM	2.20	0.9
LSC(001)/GDC/YSZ	3.83	0.3
LSC(110)/GDC/YSZ	2.71	0.4

a All thin films exhibit slightly shorter out-of-plane lattice parameters than polycrystalline LSC.

Detailed sketches of the interfaces between GDC and LSC and the layer structures of LSC in different orientations, as well as AFM images of the surfaces of three differently oriented LSC thin films, are shown in the Supporting Information. While the surfaces of the (110)- and (111)-oriented LSC thin films grown on LSGM exhibit several large domains, the RMS roughness of the surfaces of all three orientations is similar and generally very low (0.5 nm for (001), 0.5 nm for (110), and 1.0 nm for (111), with values measured on single domains for the (110)- and (111)-oriented samples).

#### HRTEM

**Figure 2:**
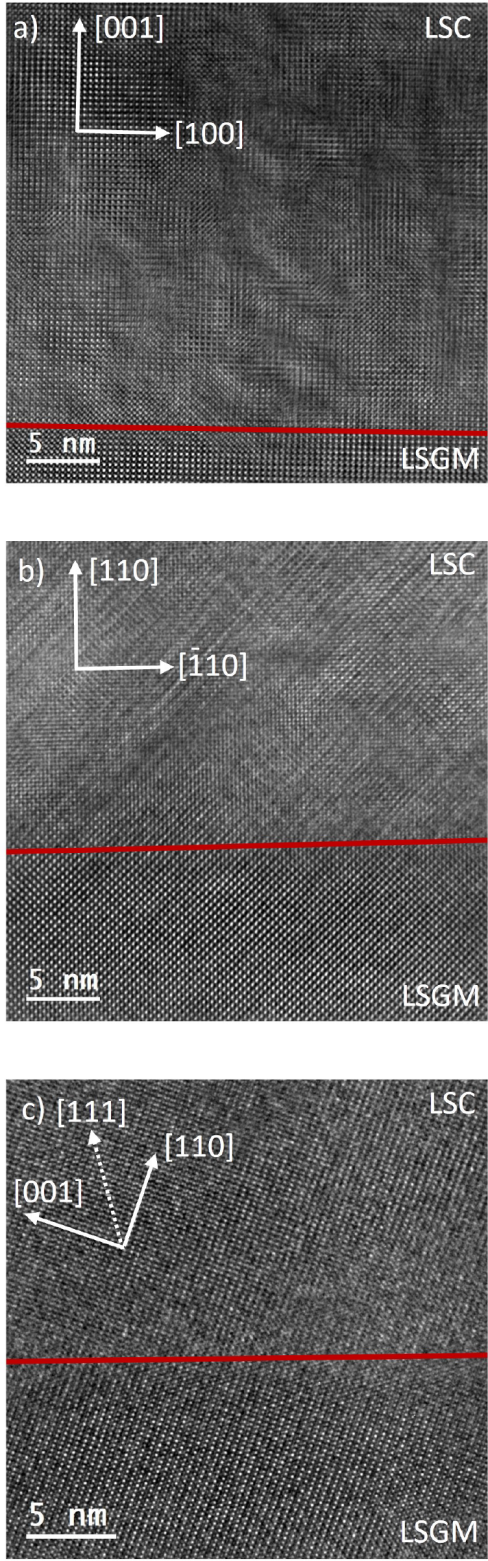
ABSF HRTEM images of the interfaces between LSGM and LSC in the orientations (a) (001) along the [010] view direction, (b) (110) along the [001] view direction, and (c) (111) along the [1̅10] view direction. Note that, due to the difficult preparation of LSGM single crystals, the (111) orientation exhibits a very large miscut of about 11°. Red lines indicate the interface positions in the images.

Figure 2 shows the average-background-subtraction-filtered (ABSF) HRTEM images of LSC thin films in the three orientations. All three images confirm that the LSC thin films grow highly oriented on the LSGM substrates. It is noteworthy that the (111) orientation exhibits a strong tilt of about 11°, which is due to a miscut of the single crystal.

When the GPA technique was applied, HRTEM was also used to evaluate the relative lattice parameter differences between LSGM and LSC. For (001) and (111), HRTEM agrees well with XRD measurements and yielded relative lattice strains of 0.53 and 1.09%; for the (110) orientation, HRTEM indicates a lower strain value of 0.03%. These results, however, need to be compared considering that the LSGM single crystals exhibit several different domains with a substantial variation of the lattice parameters. Therefore, HRTEM measurements are only capable of probing a specific region of the interface, while XRD measurements reflect the average strain of the thin film. HRTEM images of LSC grown on YSZ(001)/GDC(001) as well as YSZ(111)/GDC(111) are shown in the Supporting Information. In consideration of the surface morphology of the thin films, it is noteworthy that none of the thin films grown on LSGM exhibit grain boundaries, indicating true epitaxial growth and not a columnar, preferentially oriented growth, as is commonly the case for PLD-grown thin films. The same holds for LSC grown on YSZ/GDC(001). LSC grown on YSZ/GDC(111) is exclusively oriented in the (110) direction; however, it does exhibit in-plane rotation for different regions, indicating columnar growth.

### Electrochemical Characterization of (001)-, (110)-, and (111)-Oriented LSC Thin Films

**Figure 3:**
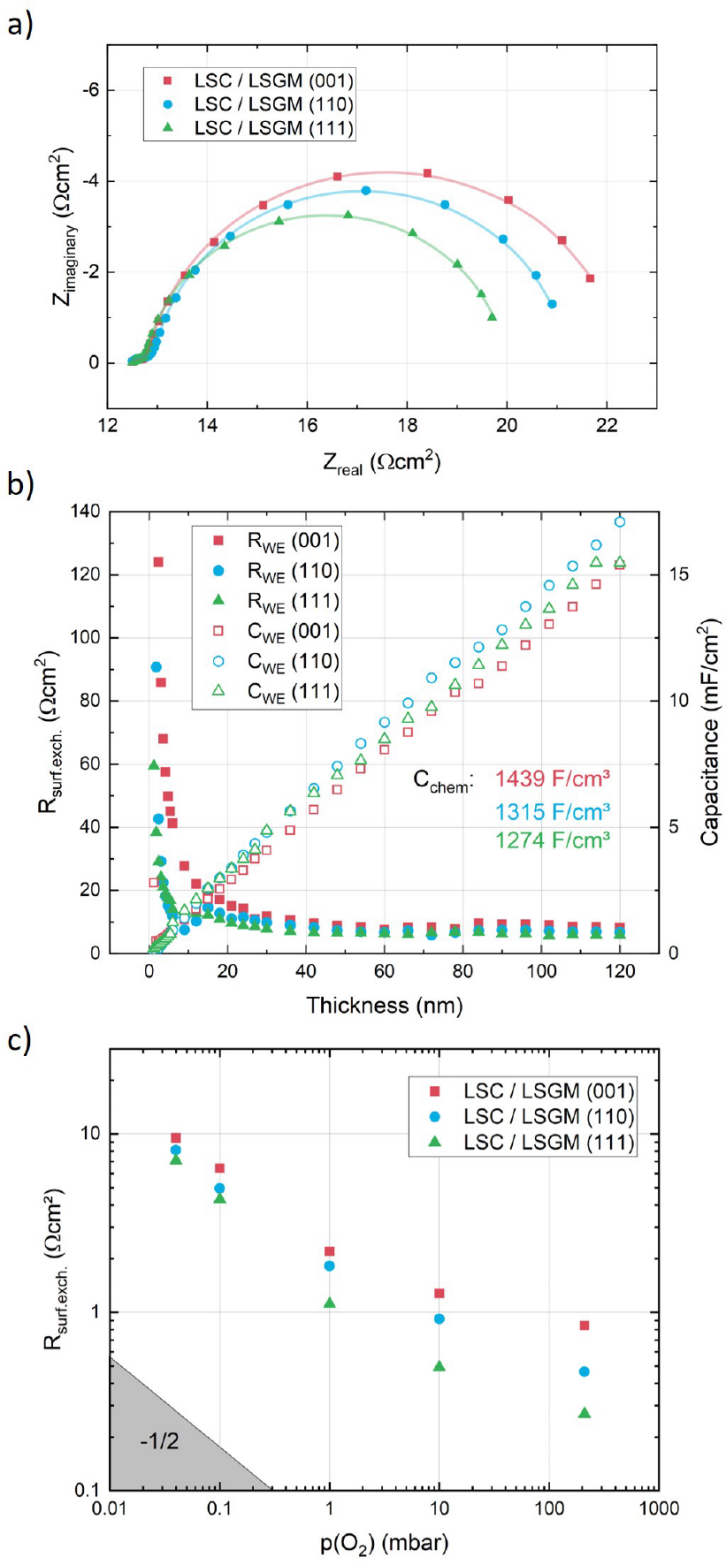
(a) Impedance curves for LSC grown on LSGM in the three different orientations, (001), (110), and (111), at 500 °C and 0.04 mbar of O_2_. The offset resistance (43 Ω electrolyte + 7 Ω grid resistance) was normalized to the sample area (0.25 cm^2^), and the surface exchange resistance was normalized to the LSC/YSZ interface area. (b) Evolution of the surface exchange resistance and chemical capacitance of the different LSC thin films during growth. (c) Oxygen partial pressure dependence of the surface exchange resistance of the three different LSC orientations, measured from low to high *p*(O_2_).

The catalytic properties of pristine LSC thin films in different orientations were examined with i-PLD, facilitating the electrochemical investigation of truly uncontaminated surfaces and their true kinetic properties. The results of these experiments are shown in Figure 3. Due to the fact that the ohmic resistance of LSGM at 600 °C only amounts to 17 Ω with ≈0.1 Ω/K changes, sensitive temperature control is not possible at these temperatures. Therefore, i-PLD of LSC grown on LSGM was performed at 500 °C at an electrolyte resistance of 43 Ω and changes of ≈0.5 Ω/K. As shown in Figure 3a, impedance spectra of growing LSC consist of an ohmic offset, a very small high-frequency feature (attributed to the interface between the electrolyte and LSC), and a dominating midfrequency semicircle, which was fitted with an R||CPE element (CPE = constant phase element).

The resistance of this feature corresponds to the surface exchange resistance. It shows a characteristic decrease with increasing film thickness (Figure 3b)^46^ because, for very thin films, the in-plane electronic conduction in the thin film limits the electron supply to the surface and thus the active area, accounting for high resistance values. After around 40 nm, the whole film surface is active and the resistance stabilizes, as was previously shown for growing LSC thin films.^46,47^

The measurements reveal a very low surface exchange resistance of 7–9 Ω/cm^2^ at 500 °C and 0.04 mbar, a factor of 10 faster than what is usually measured for LSC at these conditions.^46^ These improved oxygen exchange kinetics are only observed during i-PLD measurements and can be traced back to the aforementioned sulfate adsorbates originating from trace impurities in measurement gases, which are not present in growth conditions.^11^ This study confirms this phenomenon (see the NAP-XPS measurements below).

The results further reveal similar values for the surface exchange resistance for the three orientations, with (001) exhibiting a slightly higher value than the (110) orientation and the (111) orientation exhibiting the fastest kinetics. The same holds for the capacitative contribution of the dominating impedance feature, which describes the chemical capacitance of the LSC thin film and hence its capability to alter its oxygen nonstochiometry upon an external stimulus (such as an AC voltage). Altogether, in their pristine state and at low *p*(O_2_), the oxygen exchange kinetics of LSC between different orientations varies only slightly. For a more detailed investigation of the OER, an oxygen partial pressure variation was performed in the PLD chamber. A characteristic *p*(O_2_) dependence was observed for all three thin films, with a slope slightly below −0.5 for low oxygen partial pressures (<1 mbar) and a flattened slope at higher pressures. Similar slopes have been reported for a variety of materials in i-PLD and discussed with regard to their implications on the OER mechanism.^48^ At high oxygen partial pressures above 1 mbar, the three orientations begin to deviate, with the (001) orientation experiencing the weakest decrease of the surface exchange resistance (and, hence, the slowest oxygen exchange kinetics), followed by (110) and (111). This is possibly due to gaseous impurities at high pressures (see below).

**Figure 4:**
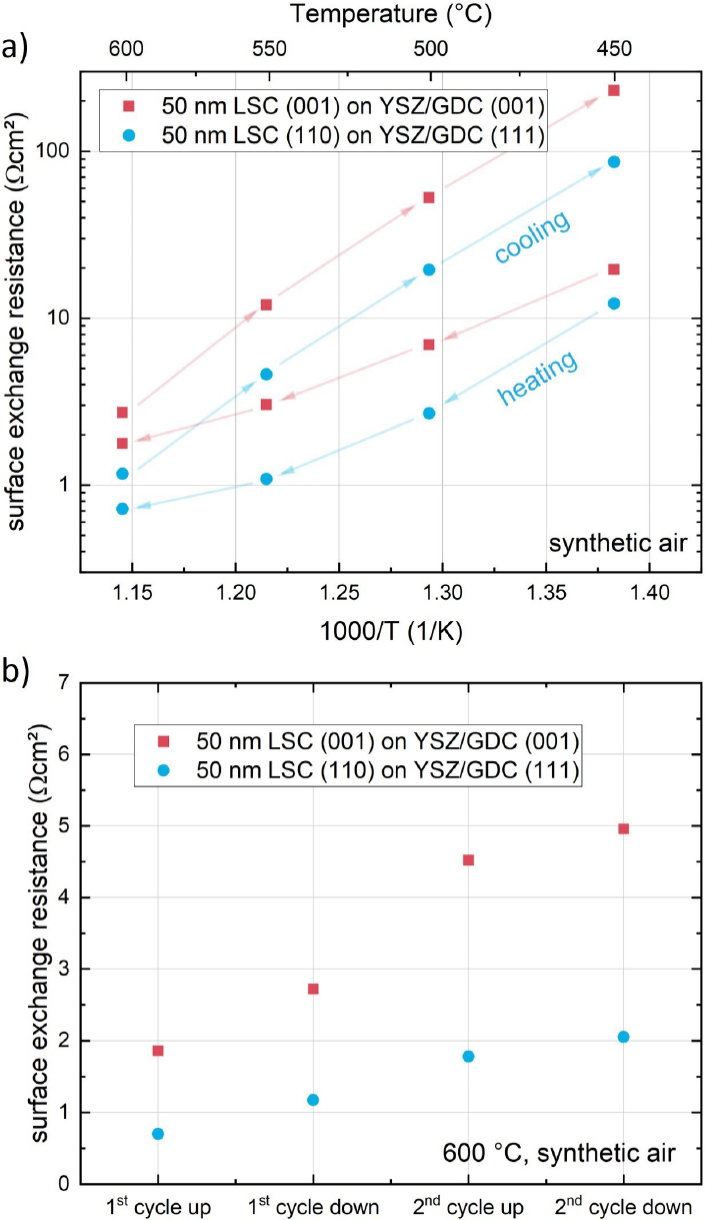
(a) *Ex situ* temperature dependence of the surface exchange resistance during the first heating cycle of (001)- and (110)-oriented LSC thin films in synthetic air. (b) Evolution of the surface exchange resistance of (001)- and (110)-oriented LSC at 600 °C during *ex situ* temperature cycling.

In addition to i-PLD investigations, the oxygen exchange kinetics of different LSC orientations were also investigated *ex situ*. Because of the limited availability of differently oriented LSGM single crystals, *ex situ* analysis was performed on LSC thin films grown on YSZ single crystals with a GDC buffer layer, facilitating the investigation of (001)- and (110)-oriented LSC surfaces (to warrant the compatibility of these different samples, the surface exchange resistance of LSC grown on different substrates was compared in i-PLD; for details, see Figure S4). Here, during measurements in synthetic air, a phenomenon similar to that in high pressures during i-PLD measurements (but at substantially higher resistance values) was observed: the catalytic activity of (001)-oriented LSC was significantly decreased compared to (110)-oriented LSC (Figure 4 a). In addition, during temperature cycling (Figure 4b), (001)-oriented surfaces experience a substantially stronger degradation than (110)-oriented surfaces. It is further noteworthy that the temperature dependence of the surface exchange resistance during the first heating cycle (Figure 4a) does not exhibit a simple Arrhenius-like behavior. At high temperatures, it appears as if accelerated degradation processes start to affect the resistance. Morphological differences might also play a role in the temporal evolution of the LSC surface because LSC grown on (111)-oriented GDC/YSZ exhibits columnar growth with in-plane rotation. In addition, we expect that the morphology changes during temperature cycling because of accelerated cation segregation and particle formation at elevated temperatures (see below).

In conclusion, electrochemical characterization of differently oriented LSC thin film surfaces yields two important results. First, in their pristine state, which was rendered accessible by using the i-PLD method, differently oriented LSC surfaces exhibit very similar oxygen exchange kinetics (comparable results were also previously obtained for LSC thin films with different grain sizes^46^). Second, as soon as the gas pressure is increased *in situ* or when samples are measured *ex situ* in ambient-pressure synthetic air, (001)-oriented surfaces perform significantly worse in comparison to other orientations. For a more in-depth investigation of this phenomenon, NAP-XPS measurements were performed.

### Surface Chemistry of Oriented LSC Thin Films

Previous i-PLD and NAP-XPS studies showed that the surfaces of different mixed conducting oxides are very susceptible to the adsorption of acidic species such as sulfur compounds, present in very low concentrations (<0.5 ppmv) even in nominally clean (5.0 purity) measurement atmospheres.^11,49^

During i-PLD measurements at low pressures, the PLD chamber acts as a getter environment, thus, it is possible to measure truly pristine surface properties. In other environments, such impurities adsorb on the surfaces already at very low gas pressures and cause a substantial decrease of the oxygen exchange kinetics. To investigate a potential correlation of sulfate adsorbates and differences between LSC orientations, three LSC thin films grown on (001)-, (110)-, and (111)-oriented LSGM substrates were investigated in the XPS chamber. Although samples were exposed to ambient air at room temperature during sample transfer, previous XPS studies have shown that LSC surfaces are primarily covered by adventitious carbonates upon exposure to air. Therefore, the samples were heated to 400 °C in 8 × 10^–6^ mbar to remove carbonates on the LSC surfaces. Afterward, the samples were heated to 600 °C and then exposed to 1 mbar of pure oxygen. Thereby, as was shown in previous studies, saturation of the surface with SO_4_^2–^ adsorbates is achieved.^11^ Afterward, the pressure was again reduced for a comparison with the sulfate-free state.

**Figure 5:**
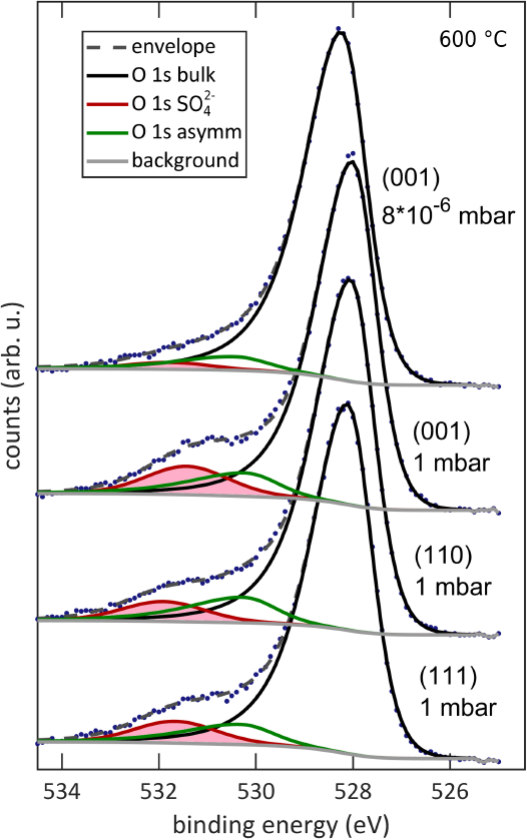
XPS measurements of the O 1s region of pristine (001)-oriented LSC at a pressure of 8 × 10^–6^ mbar and (001)-, (110)-, and (111)-oriented LSC after exposure to 1 mbar of O_2_ at a temperature of 600 °C. A second O species was necessary for an accurate fit of the asymmetry of the main O 1s peak, which we attribute to the metallic electronic structure of LSC.

**Figure 6:**
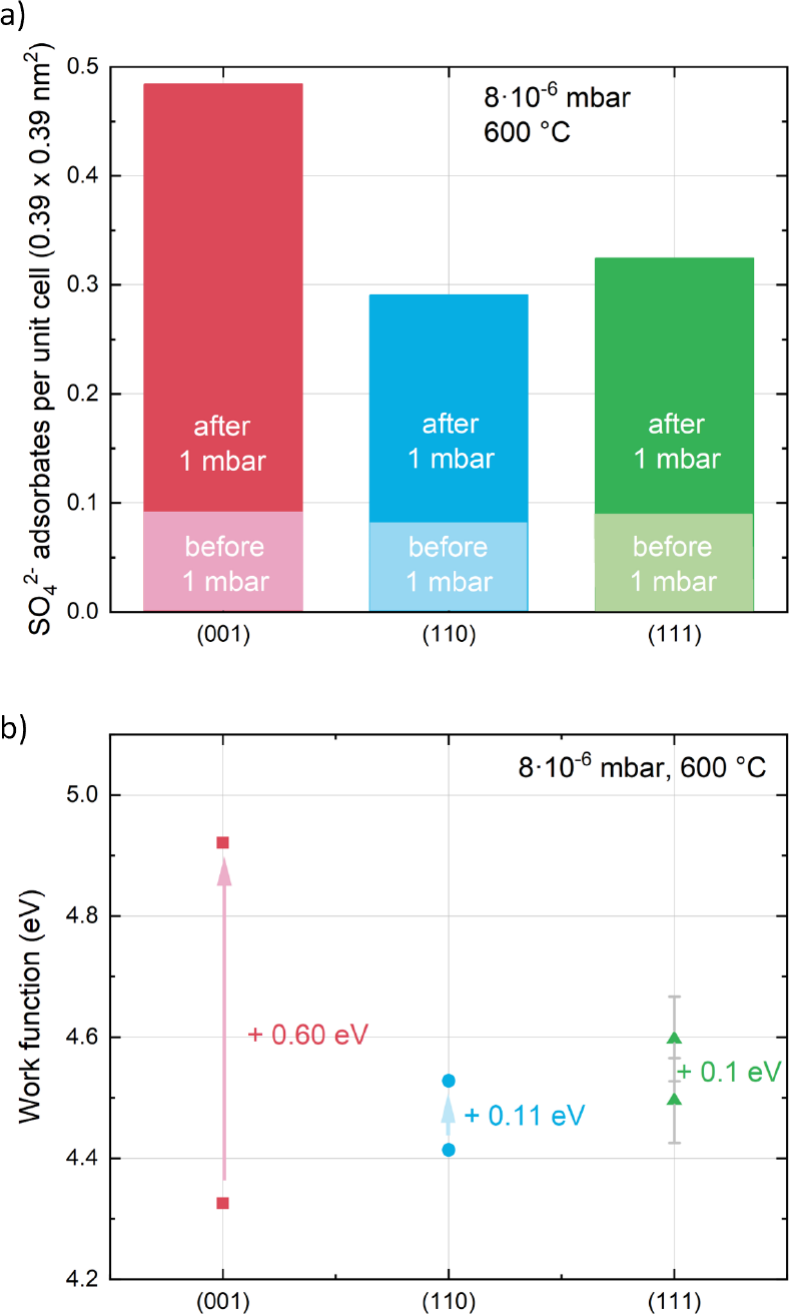
(a) Amount of SO_4_^2–^ adsorbates per LSC unit cell for (001)-, (110)-, and (111)-oriented LSC thin films before and after exposure to 1 mbar of O_2_. (b) S-induced changes of the work function of (001)-, (110)-, and (111)-oriented LSC thin films upon exposure to 1 mbar of O_2_.

The results of these measurements unambiguously show that SO_4_^2–^ adsorbates form on all three LSC orientations, clearly discernible due to the O 1s peak at 532 eV, which develops after exposure to 1 mbar of O_2_ and which has already been attributed to sulfate groups at the surface (Figure 5; more details are given in the Supporting Information).^11,50^ However, while the sulfate coverage before high *p*(O_2_) was similarly low for all three orientations, the increase of sulfate species for (001)-oriented LSC is substantially higher than for the other two orientations (Figure 6a). The SO_4_^2–^ coverage was determined from the ratio of the bulk and SO_4_^2–^ O 1s signal intensities (*I*). With an inelastic mean free path length of 1.8 nm, a mean of 4.7 unit cells or 14.1 O atoms per unit cell area are measured; consequently, the coverage equals
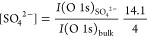
1

As an additional aspect of the surface chemistry of LSC, the work functions of the three thin films were investigated. Before exposure to 1 mbar of O_2_, the work functions of the three orientations are very similar between 4.3 and 4.5 eV, with the trend actually agreeing with the surface exchange resistance of the three orientations (Figure 6b). The work function increases significantly upon adsorbate formation, and corresponding to the higher susceptibility of (001)-oriented surfaces toward SO_4_^2–^ formation, the work function increase for LSC(001) is again significantly higher than for the other orientations. It is also noteworthy that the work function increase coincides with a decrease of the binding energy of the SO_4_^2–^-correlated O 1s species, which is due to the changing surface potential on the SO_4_^2–^-covered LSC surface.

This susceptibility toward the formation of acidic adsorbates is likely also the reason for the deviating behavior at high pressures during i-PLD. While we do not observe a continuous degradation of the oxygen exchange kinetics at these conditions, the different orientations might exhibit different equilibrium coverage with acidic adsorbates either from very small amounts of sulfur compounds present even in i-PLD conditions or from traces of CO_2_, which have recently been shown to affect the oxygen exchange kinetics during i-PLD.^51^

The combination of electrochemical and surface analytical measurements thus strongly suggests that the difference in the oxygen exchange kinetics between (001)-oriented LSC and (110)- as well as (111)-oriented LSC is connected to the orientation-dependent susceptibility toward the formation of SO_4_^2–^ (and possibly various other acidic) adsorbates and thereby induced work function changes. In the following, we will discuss this result in the context of previous literature reports as well as its relevance with regard to degradation processes on real SOFC cathode surfaces under operating conditions.

## Discussion

To truly unravel the interplay between the crystal orientation and the oxygen surface exchange kinetics, many aspects have to be considered in detail, particularly the exact *reconstruction of the surface* under operating conditions and the *detailed OER mechanism* and its rate-determining step. While both of these aspects are far from being understood, both are active areas of research, and progress is being made in both topics. In addition, this study highlights the importance of the *dynamic surface and adsorbate chemistry* for possible differences between crystal orientations.

### Surface Reconstructions

In recent studies, it has been shown that the surface of perovskite materials can exhibit a variety of different reconstructions, depending on the oxygen chemical potential and on the surface cation composition.^52−54^ In particular, polar surfaces such as the termination of (110)- and (111)-oriented perovskite surfaces can be expected to reconstruct at elevated temperatures, when the cation mobility is sufficiently high. Moreover, at high temperatures, segregation processes start to affect the surface chemistry and surface reconstructions might become even more complicated.^53^ Therefore, it is difficult to compare computational studies of ideal terminations with experimental data obtained at high temperature and deduce implications for surface orientation effects.

While an exact analysis of surface reconstructions would require sophisticated, atomic resolution scanning tunnelling microscopy, or AFM, we will assess possible surface reconstruction and morphology effects according to the available data and considering previous research. Tapping-mode AFM images (see the Supporting Information) showed that the surfaces of LSC grown on LSGM single crystals in three different orientations exhibit seemingly different surface morphologies. While the surface on (001)-oriented LSC is relatively flat on a micron scale, the surfaces of (110)- and (111)-oriented samples exhibit several large domains with large height differences of up to 8 nm, originating from LSGM single crystal domains rather than from atomic surface terraces. Focusing on these individual domains, further analysis shows that all surfaces are flat on the nanoscale (RMS roughness ≤1 nm) and that all thin films show a certain surface mosaicity. While this mosaicity is often correlated with the presence of grain boundaries, HRTEM images do not indicate classic columnar growth in the bulk; therefore, we suspect the presence of either small surface features or low-angle grain boundaries. Considering that the oxygen exchange on pristine LSC surfaces takes full advantage of the whole mixed conducting surface,^46^ such features are unlikely to contribute significantly to the oxygen exchange kinetics. Hence, our i-PLD results indicate that possibly different surface reconstructions of different crystal orientations do not have a substantial impact on the catalytic properties of LSC surfaces in their pristine state because all three orientations exhibit similar surface exchange resistances in their initial state. This initial kinetic similarity is, however, lost as soon as the surfaces get in contact with sulfate adsorbates at higher pressures.

At this point, it is also noteworthy that lattice strain is an additional factor that might come into play when discussing the oxygen exchange kinetics of differently structured thin films. It has been widely accepted that lattice strain has a substantial impact on the oxygen exchange kinetics and tensile strain accelerates the oxygen exchange.^17,19,32,55^ However, because the differently oriented thin films all exhibit similar average strain values and we do not observe an increased amount of dislocations in HRTEM indicating strain relaxation, we do not suspect a big influence of lattice strain on the variation of the oxygen exchange kinetics.

### OER Mechanism

Regarding the reaction mechanism itself, several recent studies have advanced our understanding of the reaction mechanism.^27,48,56−58^ It became clear that oxygen vacancies as well as electron transfer to oxygen adsorbates play a decisive role for the reaction rate of this complicated reaction. Considering the complex surface structures that are present on perovskite surfaces, it is possible that specific surface reconstructions exhibit different surface defect concentrations, which are beneficial for the OER, although an experimental proof for this has not been given yet and the effect on pristine surfaces again seems to be negligible.

### Adsorbate and Surface Chemistry

**Figure 7:**
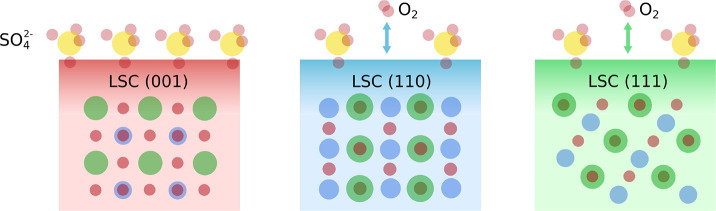
Visualization of the different susceptibilities of the three crystal orientations toward sulfate adsorbate formation.

The main experimental result of this study, a stronger susceptibility of (001)-oriented LSC surfaces toward SO_4_^2–^ adsorbate formation, however, has so far not been accounted for in this discussion (Figure 7). Because LSC thin films grown epitaxially on LSGM in all three orientations exhibit a similar surface roughness, we suspect that this susceptibility difference is tied to the nanoscopic surface reconstructions of the specific orientations. This might also include different sulfate configurations and dipole inclinations that manifest in different surface potential steps.

As was already suggested in an earlier in-depth study of the effect of SO_4_^2–^ adsorbates on mixed conducting surfaces,^11^ the very low S signal is easily overlooked in a survey scan and the O 1s species at 532 eV has only recently been assigned to SO_4_^2–^. In a further study on the orientation dependence of the oxygen exchange kinetics of La_0.8_Sr_0.2_Co_0.2_Fe_0.8_O_3−δ_,^33^ a similar O 1s species was observed and its size is roughly inversely proportional to the surface exchange coefficient for the different orientations, suggesting that the here-uncovered correlation of crystal orientation and susceptibility for acidic adsorbates is the rule rather than the exception. It is therefore clear that a deeper insight in the underlying mechanism behind acidic adsorbate formation requires exact knowledge of the surface reconstructions and compositions and the use of detailed surface analytics.

Apart from potential reasons for the different performances of specific crystal orientations, this study also deepens our understanding regarding the cause of the resistance increase upon sulfate adsorbate formation. The work function increase indicates the formation of a surface dipole and a negative charge accumulation in the adsorbate layer. This concept of surface charge redistribution upon acidic adsorption has already been brought forward in recent studies^11,51^ and suggests that sulfate adsorbates affect multiple contributions to the OER rate (e.g., adsorption barriers, adsorption energies, surface defect concentrations, and the surface potential step). It also agrees with the concept that the surface acidity is an important factor for the oxygen exchange kinetics, with SO_4_^2–^ anions increasing the acidity and strongly inhibiting the oxygen exchange on LSC surfaces.

The *ex situ* measurements presented in this study show that a higher susceptibility for acidic adsorbate formation also leads to stronger long-term degradation in synthetic air at elevated temperatures. Here, also morphological differences might play a role because (001)-oriented LSC exhibits epitaxial growth on GDC/YSZ, while (110)-oriented LSC exhibits columnar film growth. A recent study of Piskin et al.,^59^ performed on polycrystalline LSC, revealed that the surface coverage with Sr-rich precipitates on the surfaces of (001)-oriented grains after annealing is much higher than that for (110)- and (111)-oriented grains, strongly supporting the aforementioned hypothesis. In addition, large precipitates were shown to contain Sr as well as S. Supported by additional recent investigations of particle formation processes on LSC thin-film surfaces,^60^ we suggest that the SO_4_^2–^ adsorbate formation observed here is the starting point for a long-term degradation process, leading to particle formation and a strong alteration of the surface chemistry of the LSC film. Thereby, the initial differences between crystal orientations in terms of different surface reconstructions and different susceptibilities toward sulfate adsorbates might manifest in persistent performance differences under operating conditions. Similar mechanisms in terms of work function changes and particle formation might also hold for other acidic compounds, which are known to affect the performance of SOFC cathode materials (such as CO_2_, CrO_3_, or SiO_2_). Again, the formation of these particles and the underlying dynamics of segregation processes are not fully understood yet and require a systematic investigation of long-term degradation on the surfaces of different crystal orientations, which go beyond the scope of this study.

As a practical consequence of the results presented here, we suppose that the degradation of real SOFC electrodes (which is usually far less severe than that on model electrodes) is strongly influenced by the different crystal orientations present in the porous electrode. Potentially, specifically oriented and reconstructed surfaces may be much more resilient to the adsorption of acidic, gaseous species than others and might dominate the oxygen surface exchange on the surface of porous electrodes.

## Conclusions

The oxygen exchange kinetics and the surface chemistry of LSC were investigated by means of i-PLD and with NAP-XPS. LSC thin films, grown epitaxially on LSGM single crystals in different orientations, initially exhibit very similar oxygen exchange kinetics. However, during *in situ* measurements in high *p*(O_2_) or in *ex situ* measurement setups, (001)-oriented LSC is afflicted by a stronger performance degradation than the other orientations. XPS measurements trace this phenomenon back to a different susceptibility toward sulfate adsorbate formation. SO_4_^2–^ adsorbates accumulate significantly faster on the surface of (001)-oriented LSC thin films. In addition, the work function of LSC surfaces upon sulfate adsorbate formation increases substantially, again with the strongest increase observed on LSC(001). The effect of SO_4_^2–^ adsorbates on the oxygen exchange kinetics of LSC agrees with the concept of surface acidity and the underlying processes are tied to a surface dipole formation and a redistribution of negative charge toward the sulfate adsorbate layer. The results further suggest that acidic adsorption is a strong driver for short-term and long-term degradation and might explain previously observed differences in the oxygen exchange kinetics between different crystal orientations.

## Supporting Information Available

Sketches of layer orientations, HRTEM images of LSC grown on YSZ/GDC, AFM images of oriented LSC thin films, comparison of surface exchange values during i-PLD, and XPS details for different LSC thin films (PDF)
