# Supplementary material for: Crystal-Orientation-Dependent Oxygen Exchange Kinetics on Mixed Conducting Thin-Film Surfaces Investigated by In Situ Studies
Source: ACS Appl Energy Mater. 2023 Jun 13;6(12):6712–20. doi: 10.1021/acsaem.3c00870 (PMC10301866; doi:10.1021/acsaem.3c00870)
Supplement: Supplementary file 1 — ae3c00870_si_001.pdf [file ae3c00870_si_001.pdf]

Supporting Information

Crystal orientation dependent oxygen exchange kinetics on mixed conducting thin film surfaces investigated by in-situ studies

Matthäus Siebenhofer[1,2]*, Christoph Riedl[2], Andreas Nenning[2], Sergej Raznjevic[3], Felix Fellner[2], Werner Artner[4], Zaoli Zhang[3], Christoph Rameshan[5], Jürgen Fleig[2], Markus Kubicek[2]

[1]*Centre for Electrochemistry and Surface Technology, CEST, Wr. Neustadt, Austria*
[2]*Institute of Chemical Technologies and Analytics, TU Wien, Vienna, Austria*
[3]*Erich Schmid Institute of Materials Science*
[4]*X-Ray Center, TU Wien, Vienna, Austria*
[5]*Chair of Physical Chemistry, Montanuniversität Leoben, Leoben, Austria*

***Corresponding author**: matthaeus.siebenhofer@tuwien.ac.at

.

S.I. 1. Layer Orientations and Interface Structures

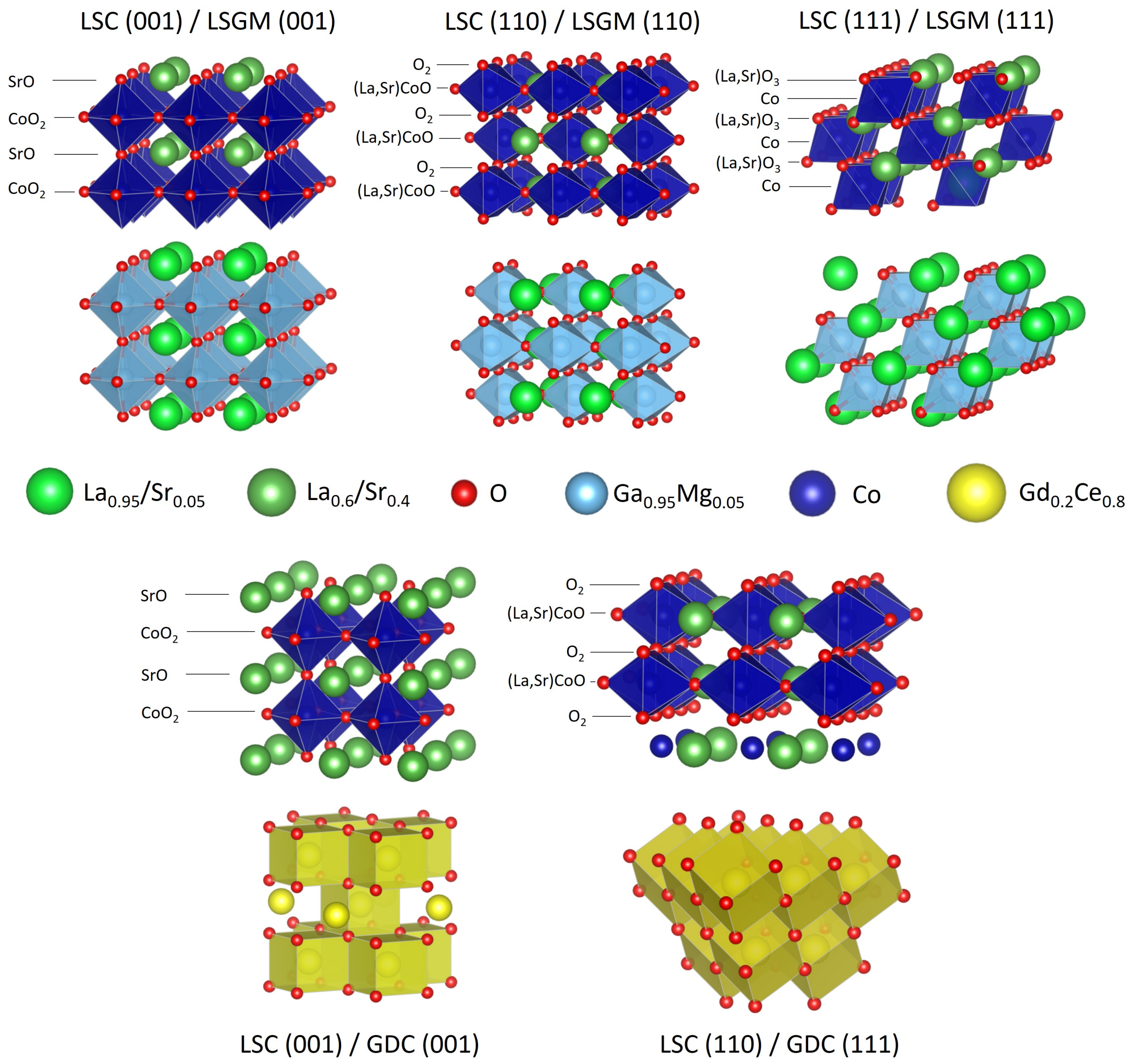

Figure S 1. Sketches of LSC grown in different orientations on LSGM (001), LSGM (110), LSGM (111), GDC (001) and GDC (111). The different layers of LSC are indicated together with the elements they contain.

S.I. 2. LSC grown on YSZ/GDC

HRTEM images of LSC grown on (001) and (111) oriented YSZ with a GDC buffer layer reveal epitaxial growth for the (001) direction with no indications of grain boundaries. Signs of a brownmillerite phase are visible, which fade around 8-10 nm from the interface. For (111) oriented YSZ, the GDC buffer layer again grows epitaxially, however, LSC exhibits different domains, indicating columnar growth with in-planed rotated grains.

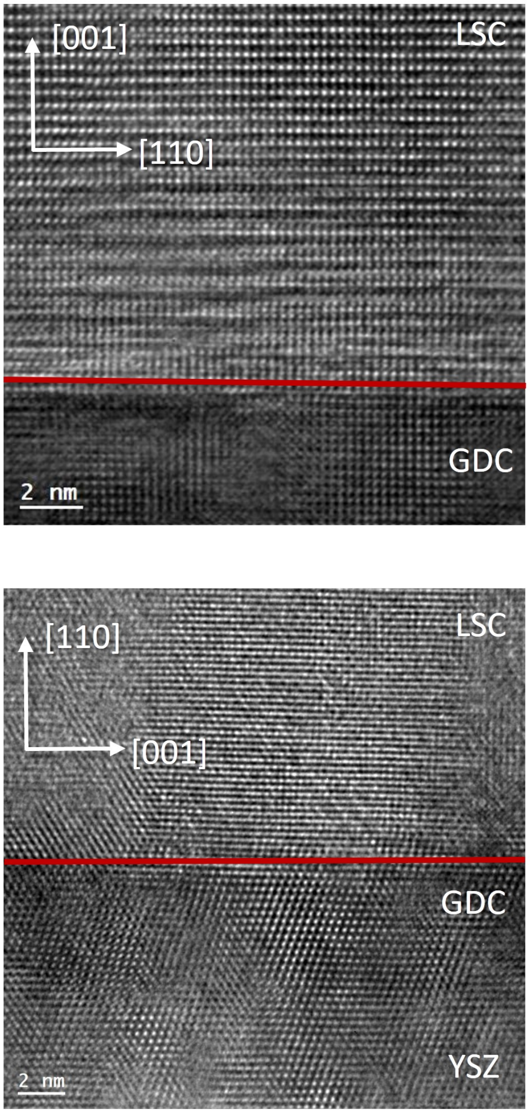

Figure S 2. HRTEM images of LSC grown on YSZ/GDC (001) and (111). Brownmillerite phases are visible near the interface for (001) oriented LSC, domains with different in-plane orientation are visible for (110) oriented LSC.

S.I. 3. Surface Morphology

The surface morphology of LSC grown in three orientations on LSGM (001), LSGM (110) and LSGM (111) directly after deposition. The macroscopic structures that can be seen on the surface originate from twin structures and crystal domains in the substrate.

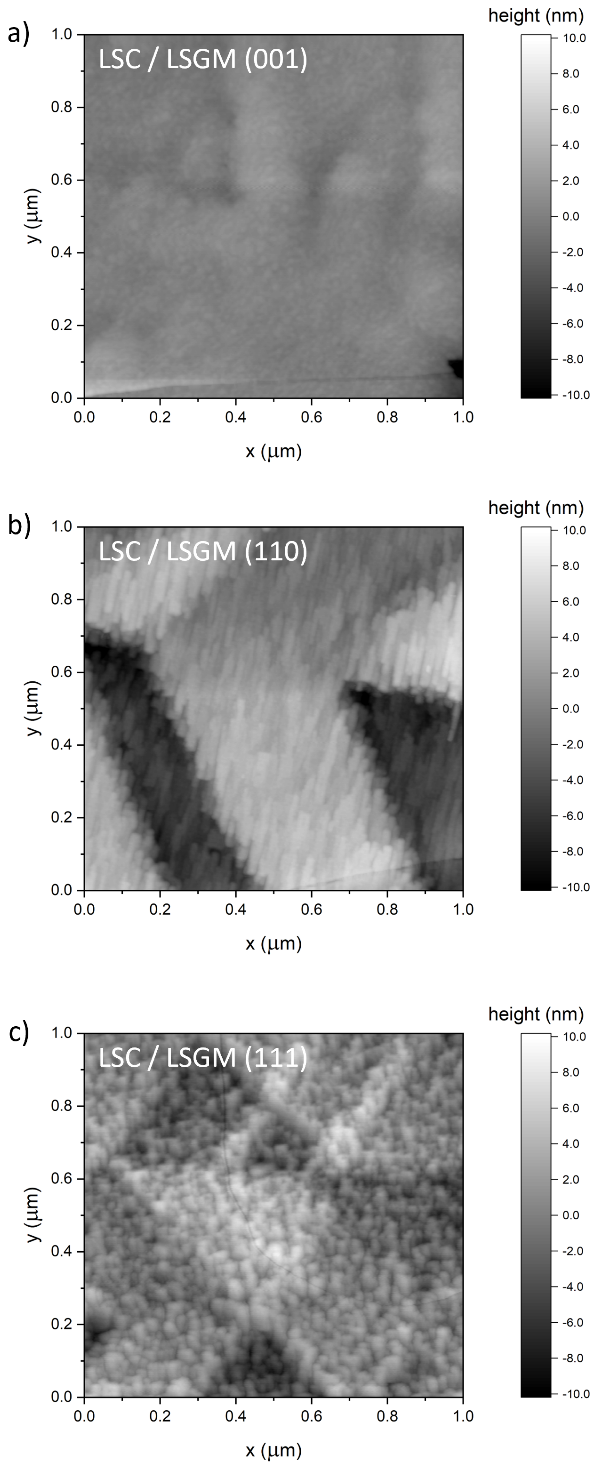

Figure S 3. AFM images of LSC grown in different orientations on LSGM (001), LSGM (110) and LSGM (111).

S.I. 4. Sample comparison

To warrant compaptibility of in-situ and ex-situ measurements, the surface exchange resistance of 40 nm thick LSC thin films grown on different substrates at 600 °C during i-PLD measurements in 0.04 mbar O_2 atmosphere was compared. Average resistance values and the corresponding standard errors are shown for several measurements (6 for LSC grown on YSZ, 3 for LSC grown on GDC/YSZ and 2 for LSC grown on LSGM). All three thin films exhibit similar surface exchange resistance values with the fastest kinetics observed for LSC on LSGM . We strongly suspect that this is caused by the comparatively high tensile strain for LSC grown on LSGM which is known to accelerate oxygen exchange[1].

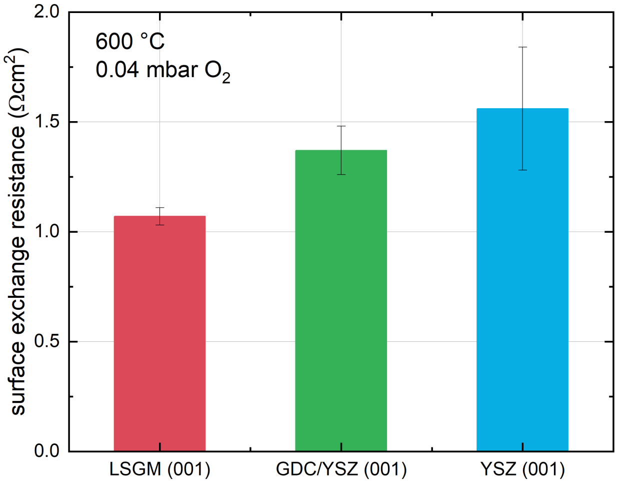

Figure S 4. Comparison of surface exchange resistance values measured by *i*-PLD for LSC grown on LSGM (001), LSC grown on GDC/YSZ (001) and LSC grown on YSZ (001). All measurements were performed at 600 °C and in 0.04 mbar O_2

S.I. 5. XPS details I

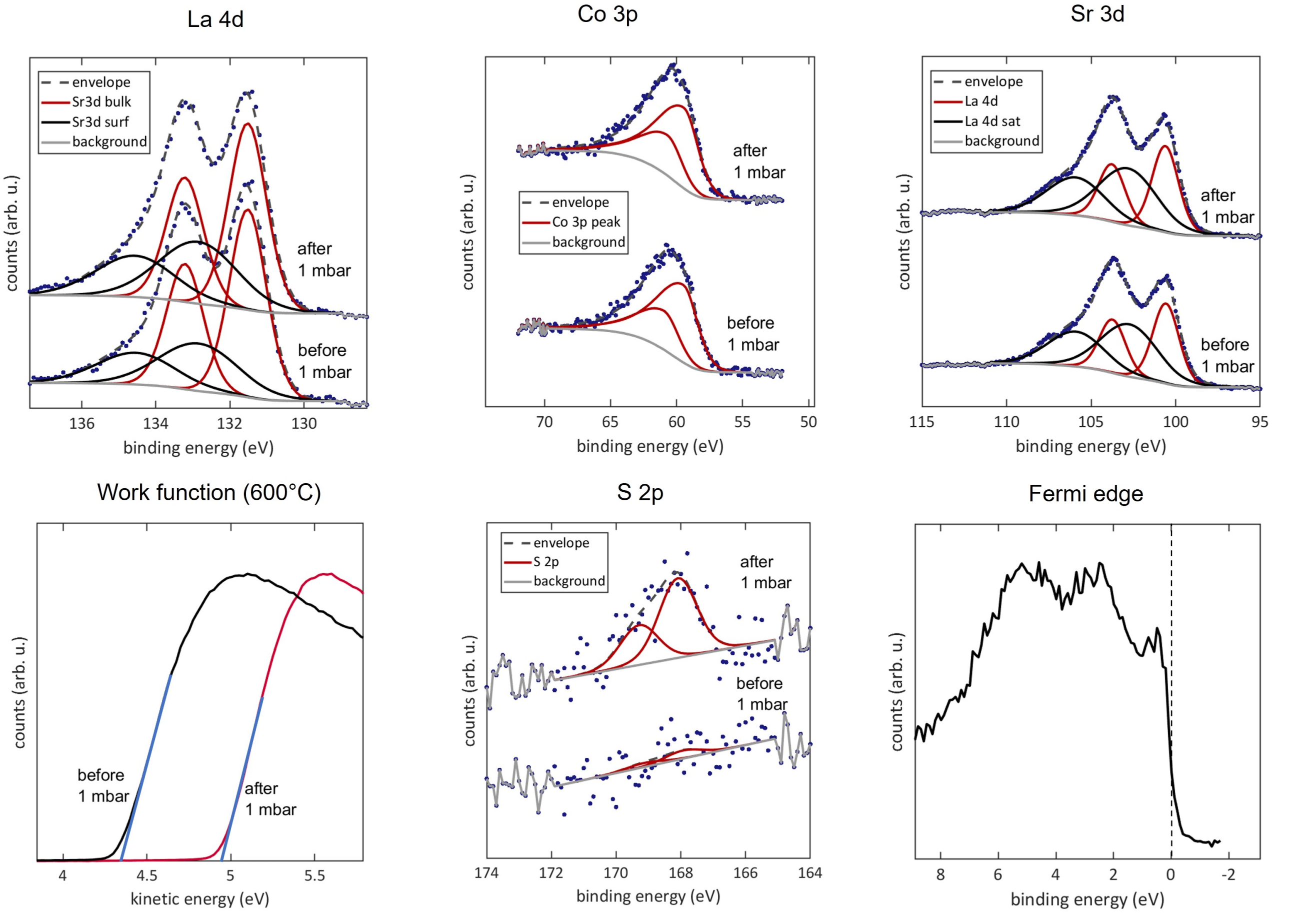

Figure S 5. First row: La 4d, Co 3p and Sr 3d signals of a (001) oriented LSC thin film grown on a (001) oriented LSGM substrate, measured during NAP-XPS in $8{\cdot}10^{-6}$ mbar O_2 at 400 °C, before and after exposure to 1 mbar O_2 at 600 °C. Second row: work function increase upon exposure to 1 mbar O_2, S 2p signal before and after exposure to 1 mbar O_2, Fermi edge region of LSC after exposure to 1 mbar O_2.

S.I. 6. XPS details II

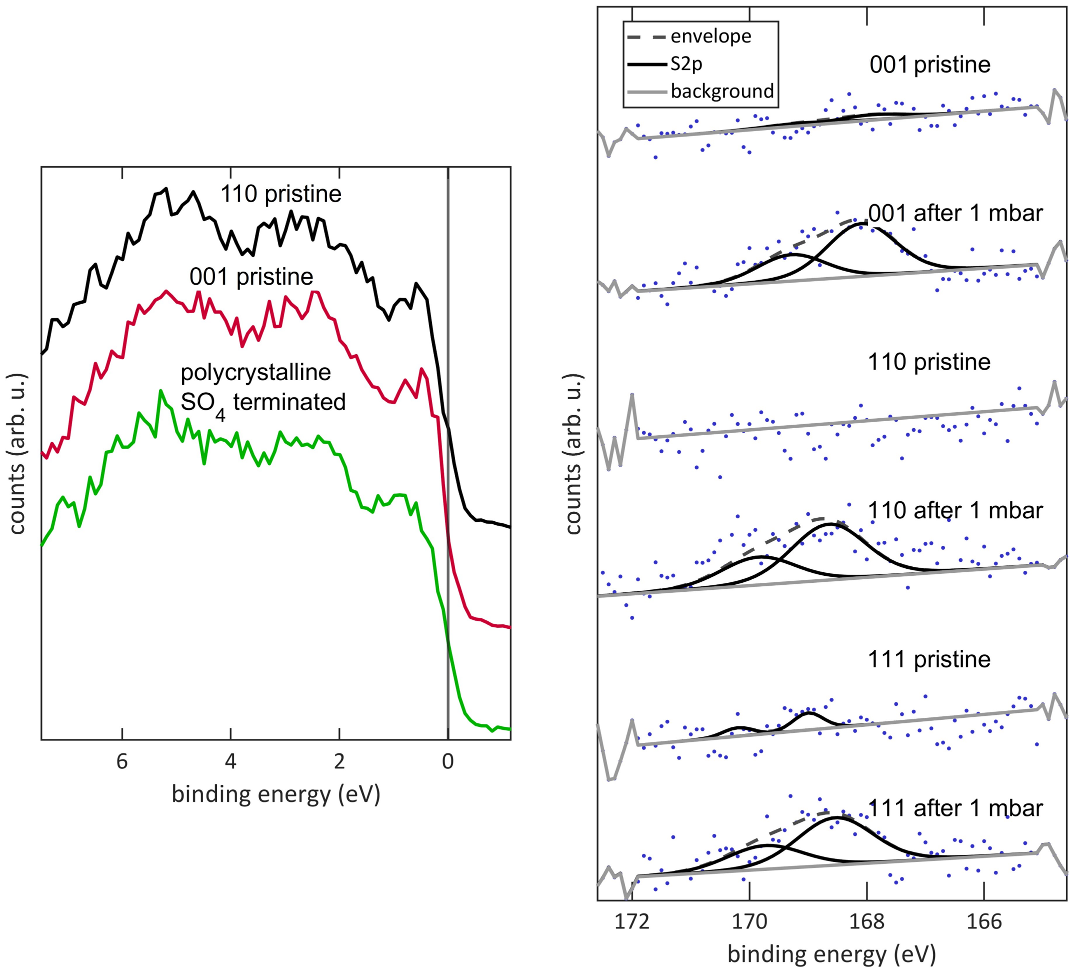

Figure S 6. left: Fermi edge region of pristine, 001-oriented and 110-oriented LSC thin film surfaces, as well as of a polycrystalline (preferentially 110-oriented) surface for comparison. All three measurements show very similar Fermi edge regions, indicating the similar band structure in all three cases. right: S2p region for different LSC orientations before and after exposure to 1 mbar O_2 and subsequent sulphate adsorbate formation. It is noteworthy that quantification of SO_4 species via the O1s area is much more precise, as the signal intensity is 8 times as high as for S2p (2x cross section and 4x stoichiometric factor).

S.I. 7. LSC cation composition and surface reconstructions

When we compare the surface compositions of differently oriented LSC films we find that all surfaces right after deposition exhibit more Sr and La, and less Co (36-39 %), compared to 50 % in the nominal stoichometry. Substantial Co depletion of the LSC surface due to A-site cation segregation was already observed before in literature. Interestingly, the surface composition is very stable regarding the different substrate orientations and atmospheric annealing steps - presumably this is linked to the short 1 mbar annealing time of 20-30 minutes - for longer annealing times, strong Sr segregation on polycrystalline LSC films was observed previously in literature[2]. Possibly, the atomic composition of the surface layer does not correspond to a perovskite bulk-plane, and it also might be similar for all three film orientations, accounting for the consistent work functions and XPS compositions.

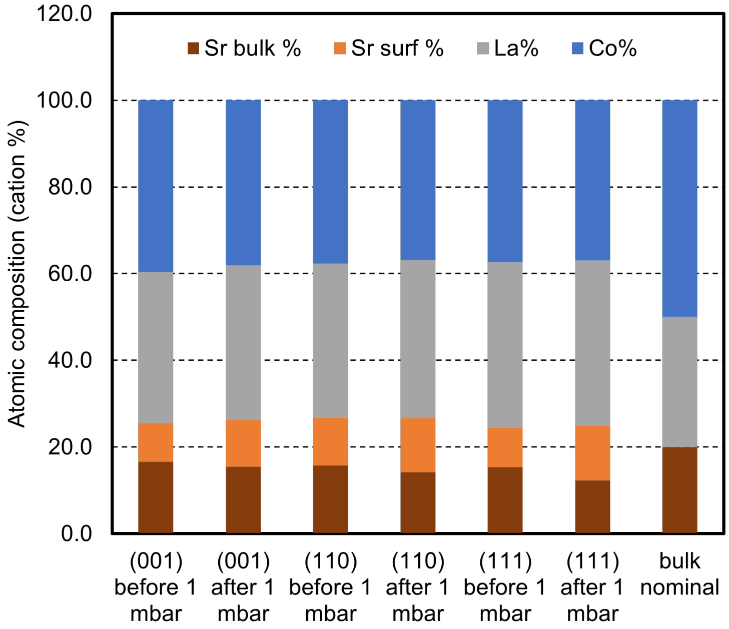

Figure S 7. Composition of the topmost 2.2 nm of differently oriented LSC thin films; Peak areas were normalized to 100%, and corrected for the inelastic mean free path (IMFP) length (KE exponent 0.7) and experimentally derived sensitivity factors by Christ[3]. For better quantification, peaks with similar kinetic photoelectron energy (La 4d, Sr 3d, Co 3p) were chosen, in order to minimize the effects of IMFP variation and the analyser transmission function.

References

(1) Rupp, G. M.; Kubicek, M.; Opitz, A. K.; Fleig, J. *ACS applied energy materials* **2018**, *1*, 4522–4535, DOI: 0.1021/acsaem.8b00586.

(2) Opitz, A. K.; Rameshan, C.; Kubicek, M.; Rupp, G. M.; Nenning, A.; Götsch, T.; Blume, R.; Hävecker, M.; Knop-Gericke, A.; Rupprechter, G.; Klötzer, B.; Fleig, J. *Topics in catalysis* **2018**, *61*, 2129–2141.

(3) Brundle, C. R.; Crist, B. V. *Journal of Vacuum Science & Technology A: Vacuum, Surfaces, and Films* **2020**, *38*, 041001.
